# Altered metabolism by autophagy defection affect liver regeneration

**DOI:** 10.1371/journal.pone.0250578

**Published:** 2021-04-29

**Authors:** Yi Chen, Zhiwei Xu, Yanli Zeng, Junping Liu, Xuemei Wang, Yi Kang

**Affiliations:** 1 Clinical Research Service Center, Henan Provincia People’s Hospital, Zhengzhou University People’s Hospital, Henan Province, Zhengzhou, China; 2 Department of Infectious Diseases, Henan Provincia People’s Hospital, Zhengzhou University People’s Hospital, Henan Province, Zhengzhou, China; 3 Department of Traditional Chinese Medicine, Henan Provincia People’s Hospital, Zhengzhou University People’s Hospital, Henan Province, Zhengzhou, China; National Institutes of Health, UNITED STATES

## Abstract

Autophagy is the primary intracellular catabolic process for degrading and recycling long-lived proteins and damaged organelles, which maintains cellular homeostasis. Autophagy has key roles in development and differentiation. By using the mouse with liver specific knockout of autophagy related gene 5 (*Atg5*), a gene essential for autophagy, we investigated the possible role of autophagy in liver regeneration after 70% partial hepatectomy (PHx). Ablation of autophagy significantly impaired mouse liver regeneration, and this impairment was associated with reduced hepatocellular proliferation rate, down-regulated expression of cyclins and tumor suppressors, and increased hepatocellular apoptosis via the intrinsic apoptotic pathway. Ablation of autophagy does not affect IL-6 and TNF-α response after PHx, but the altered hepatic and systemic metabolic responses were observed in these mice, including reduced ATP and hepatic free fatty acid levels in the liver tissue, increased glucose level in the serum. Autophagy is required to promote hepatocellular proliferation by maintaining normal hepatic and systemic metabolism and suppress hepatocellular apoptosis in liver regeneration.

## Introduction

Autophagy is a catabolic process by which cells remove protein aggregates and damaged organelles. During autophagy, double-membrane vesicles, known as autophagosomes, are generated to sequester part of the cytoplasm. These autophagosomes will subsequently fuse with lysosomes, and their cargos will be digested by lysosomal enzymes for recycling. By using yeast genetics, more than 30 genes that are involved in autophagy have been identified. Many of the mammalian homologues of these autophagy (*Atg*) genes have also been isolated [1, 2]. Autophagy plays an important role in maintaining cellular homeostasis and Autophagy has key roles in development and differentiation [1, 2].

The liver possesses a unique capacity to return to a constant size within a short period after injury or partial hepatectomy (PHx). It has previously been demonstrated that the progress of liver diseases is dependent on repeated rounds of hepatocyte apoptosis and proliferation [3]. Hepatocyte proliferation is responsible for liver regeneration following two-thirds PHx. Liver regeneration after PHx is a multi-step process that include cytokines, growth factors, and metabolic networks [4–6]. Tumor necrosis factor alpha (TNF-α) and interleukin- 6 (IL-6), together with their downstream transcription factors STAT3 and nuclear factor kappa B (NF-κB), are the most important initiating factors in the regenerative response [7–10]. Activation of the initiating factors result in DNA synthesis, cell replication, and increase in cell size during the process of liver regeneration [4]. These initiating factors also allow the liver maintain its essential metabolic functions over several days after PHx [11, 12]. These initiating factors prime hepatocyte enter into the cell cycle (G0 to G1). Cell cycle progression is then driven by growth factors, which override a restriction point in late G1, then enter into S phase associated with other factors, such Rb family member p107 and cyclins A, D, and E, and cdk4/cyclin D and cdk2/cyclin E complexes [13–15]. HGF and EGF receptor (EGFR) ligand family are key growth factors that drive hepatocyte cell cycle progression during liver regeneration [16, 17]. Activation of HGF and *c-met*, the gene for HGF receptor, up-regulate ERK1/2 expression, which lead to DNA replication and hepatocyte proliferation [18, 19]. The EGF receptor ligand family, including EGF, transforming growth factor alpha (TGF-α), heparin-binding EGF-like growth factor (HBEGF), and amphiregulin, have different but overlapping effect on liver regeneration [17].

After experimentally induced hepatic injure, the metabolic demands on the liver regeneration are immense. The hepatic metabolism is rapidly and specifically altered in response to the loss of liver mass; continue to generate an adequate systemic energy to meet the requirement of DNA replication and cell division [20]. Any alterations that change normal metabolic response to liver proliferation should impair liver regeneration.

As autophagy has key roles in development and differentiation, we suspect that autophagy is important for liver regeneration. In this study, we used mice with liver specific knockout of *Atg5* gene to study the role of autophagy in liver regeneration after 70% partial hepatectomy (PHx). We found that abolishing the expression of Atg5 significantly impaired mouse liver regeneration. This effect was result of alteration of metabolic responses in the L-Atg5-KO mice comparing with Atg5-WT mice, including damaged mitochondrial, reduced ATP level in the liver tissue, reduced total protein, albumin level, increased ALT and glucose level in the serum. These finding indicated that autophagy has key role in liver remodel and reorganizing during liver regeneration.

## Materials and methods

### Production of L-Atg5-KO mice

C57BL/6 mice with liver-specific knockout of Atg5 were generated by crossing mice carrying the floxed Atg5 gene (Atg5f/f) with mice carrying the Mx1-Cre gene, which expresses Cre recombinase under control of an Mx promoter (Mx-Cre). Both strains of mice were purchased from The Jackson Laboratory (Bar Harbor, ME). Cre expression in these crossbreeding produced mice was induced by injection with 300 μL polyinosinic acid/polycytidylic acid (Sigma-Aldrich, St. Louis, MO) at a concentration of 1 mg/mL in water, three times at 48- hour intervals. Mice were maintained in a room with alternating 12-hour light/dark cycles. Our mouse studies were conducted in accordance with the Guide for the Care and Use of Laboratory Animals of the mouse and approved by the Life Science Ethics Committee of Zhengzhou University.

### Partial hepatectomy

70% partial hepatectomy (PHx) was performed on eight-weeks old male mice as described by Green and Puder [21]. Briefly, male mice were weighed and anesthetized with ether prior to the surgery. The midline laparotomy was then performed, and right medial, left medial, and left lateral lobes of the liver were removed. The resected liver weight was also measured. Mice were sacrificed at multiple time points after surgery and the regenerating liver was harvested, weighed, and used for immunohistochemistry and other studies.

### Anaesthesia and postoperative management

The inhaled anesthetic is administered using a commercial anesthesia system (Harvard Apparatus, MA, USA, Cat:726421). The mouse is placed into an induction chamber (Harvard Apparatus, MA, USA, Cat: 727110) supplied with 2% isoflurane and oxygen. Isoflurane and oxygen with a flow rate of 5 L/min are used in the induction phase and 0.5–2.0 L/min in the maintenance phase. The injectable anesthetics used are ketamine (80–100 mg/kg) and xylazine (5–10 mg/kg). Analgesic way used buprenorphine, 0.02–0.05 mg/kg, by the way of subcutaneous injection once every 6–12 h for at least 48 h after surgery. Meloxicam, 1–2 mg/kg. Orally administered, once a day. After surgery a warmer was needed to be used to maintain the body temperature immediately until the mice has completely recovered from anesthesia as anesthesia can reduce animal body temperature. For partial hepatectomy, the post-operation observation was performed once every 2 h for 12 h after the surgery and thereafter once every 4 h. An analgesic agent was given intramuscularly every 12 h for 5 days after surgery. If aseptic techniques are followed, antibiotics are not necessary. If an infection develops, gentamicin can be given intramuscularly at a dosage of 5 mg/kg for 5 days. Mice should recover within minutes after surgery. No mortality of the animal will be observed if the PHx is performed correctly.

### Immunoblotting analysis

Liver tissues were homogenized in the RIPA solution (10 mM Tris-HCl, pH 7.0, 150 mM NaCl, 1% Triton X-100, 1% sodium deoxycholate, and 0.1% sodium dodecyl sulfate) and, after a brief centrifugation to remove cell debris, the protein concentrations were determined by Bradford BCA (Biorad) and subjected to immunoblot analysis.

### Antibodies

The primary antibodies used in this study included the rabbit anti-Atg5 antibody (Cell signaling, cat:12994), rabbit anti-LC-3 antibody (MBL, cat: PM036), rabbit anti-p62 antibody (Cell Signaling, cat:5114), rabbit anti-actin antibody (Abcam, cat: ab8227), rabbit anti-Beclin-1 antibody (Abcam. ab51031), rabbit anti-Cyclin-D1 (Cell Signaling, cat:2978), rabbit anti-Cyclin-A2 (Cell Signaling, cat:4656), rabbit anti-Cyclin-E1 (Cell Signaling, cat:4129), rabbit anti-Cyclin-B1 (Cell Signaling, cat:4138), rabbit anti-p53 (Cell Signaling, cat:2524), rabbit anti-p21 (Abcam, ab7960), rabbit anti-p27 (Abcam, ab7961), rabbit anti-Caspase 8 (Cell Signaling, cat:9496), rabbit anti-Caspase 9 (Cell Signaling, cat:9509), rabbit anti-Caspase 3 (Cell Signaling, cat:9662), rabbit anti-Cytochrom C (Cell Signaling, cat:4272), rabbit anti-COX IV (Cell Signaling, cat:4844).

### Immunohistochemistry

Paraffin-embedded liver tissue sections were stained for proliferating cell nuclear antigen (PCNA). Briefly, liver tissue sections were treated with 0.01M sodium citrate in the microwave oven for 10 minutes for epitope retrieval, and then blocked using the goat serum. Tissue sections were incubated with the primary antibody and the secondary biotinylated affinity-purified goat anti-rabbit IgG (Invitrogen, cat: 32460), and the staining was then developed using the avidin-conjugated horseradish peroxidase (HRP) with diaminiobenzidine (DAB) as the substrate (Ultra-sensitive ABC Peroxidase Staining Kit, Thermo). Rabbit polyclonal to PCNA was purchased from Abcam (cat: ab18197).

### TUNEL assay

The TUNEL assay was performed using the Roche In Situ Cell Death Detection kit following the manufacturer’s protocol.

### Real-time RT-PCR analysis of IL-6 and TNF-α mRNAs

Liver tissues were homogenized in Trizol (Invitrogen) and total RNA was isolated following the manufacturer’s protocol. The cDNA synthesis was performed using 1 μg RNA, the random hexamer primer (Roche), and the MMLV Reverse Transcriptase kit (Applied Biosystems). The real-time PCR was performed using the Invitrogen Power SYBR® Green PCR Master Mix and the Applied Biosystems 7500 Fast Real-Time PCR System. The primers used for RT-PCR were as follows: IL-6, forward: 5′-GTTCTCTGGGAAATCGTGGA-3′ and reverse: 5′-TGTACTCCAGGTAGCTATGG-3; TNF-α, forward: 5’-CCCACGTCGTAGCAAACCAC-3’ and reverse, 5’-CGTAGTCGGGGCAGCCTTGTC-3’; GAPDH, forward: 5’-TGAACGGGAAGCTCACTGG-3’, reverse: 5’-TCCACCACCCTGTTGCTGTA-3’. PCR reactions were carried out in a total volume of 25 μl. The reaction mixture contained 2× SYBR Mix (12.5 μl) from the kit, 0.4 pmol/μl from each primer and 4U Taq DNA polymerase, and 1 μl cDNA. The 2× SYBR Mix contained the PCR buffer, Mg^2+^, dNTP mixture, and SYBR Green®. The thermal cycling condition included an initial denaturation step at 95°C for 10 minutes and 40 reaction cycles consisting of a denaturation step at 95°C for 15 seconds and an annealing/elongation step at 60°C for 60 seconds. Fluorescent measurements were taken at the annealing/elongation step. Each sample was run in duplicates and mean Ct values were used for further calculations.

### Oil-red O staining

Liver tissues, which were frozen in OCT compounds, were cut at 5 μm, mounted on slides and allowed to dry for 1–2 hours. The sections were fixed with 10% formalin for 10 minutes and then the slides were rinsed with PBS (pH 7.4). After air dry, the slides were placed in 100% propylene glycol for 2 minutes, and stained in 0.5% oil red O solution in propylene glycol for 30 minutes. The slides were transferred to an 85% propylene glycol solution for 1 minutes, rinsed in distilled water for 2 changes, and processed for hematoxylin counter staining.

### Hepatic ATP assay

50 mg liver tissue was homogenized in ATP assay buffer. Centrifuge the samples at 13,000×g for 10 minutes to remove insoluble material. Collect the sup and measure the ATP level according to manufacturer’s instruction (Gene Tex, cat: YS02564BI).

### Hepatic free fatty acid assay

10 mg liver tissue was homogenized in 200 μl of a 1% (w/v) Triton X-100 in chloroform solution. Centrifuge the samples at 13,000×g for 10 minutes to remove insoluble material. Collect the organic phases and air dry at 50°C to remove chloroform. Vacuum dry for 30 minutes to remove trace chloroform. Dissolve the dried lipids in 200 μl of free fatty acid assay buffer by vortexing extensively for 5 minutes. The solution was used to measure free fatty acid level according to manufacturer’s instruction (Sigma-Aldrich, cat: MAK044).

### Serum glucose assay

4 μl serum was diluted in 96 μl ddH2O. The diluted sample was deproteinized using 10KD Spin column (Abcam, cat:93349). The solution was used to measure glucose level according to manufacturer’s instruction (Abcam, cat:65333).

## Results

### Generation of liver-specific Atg5-deficient mice

**Fig 1:**
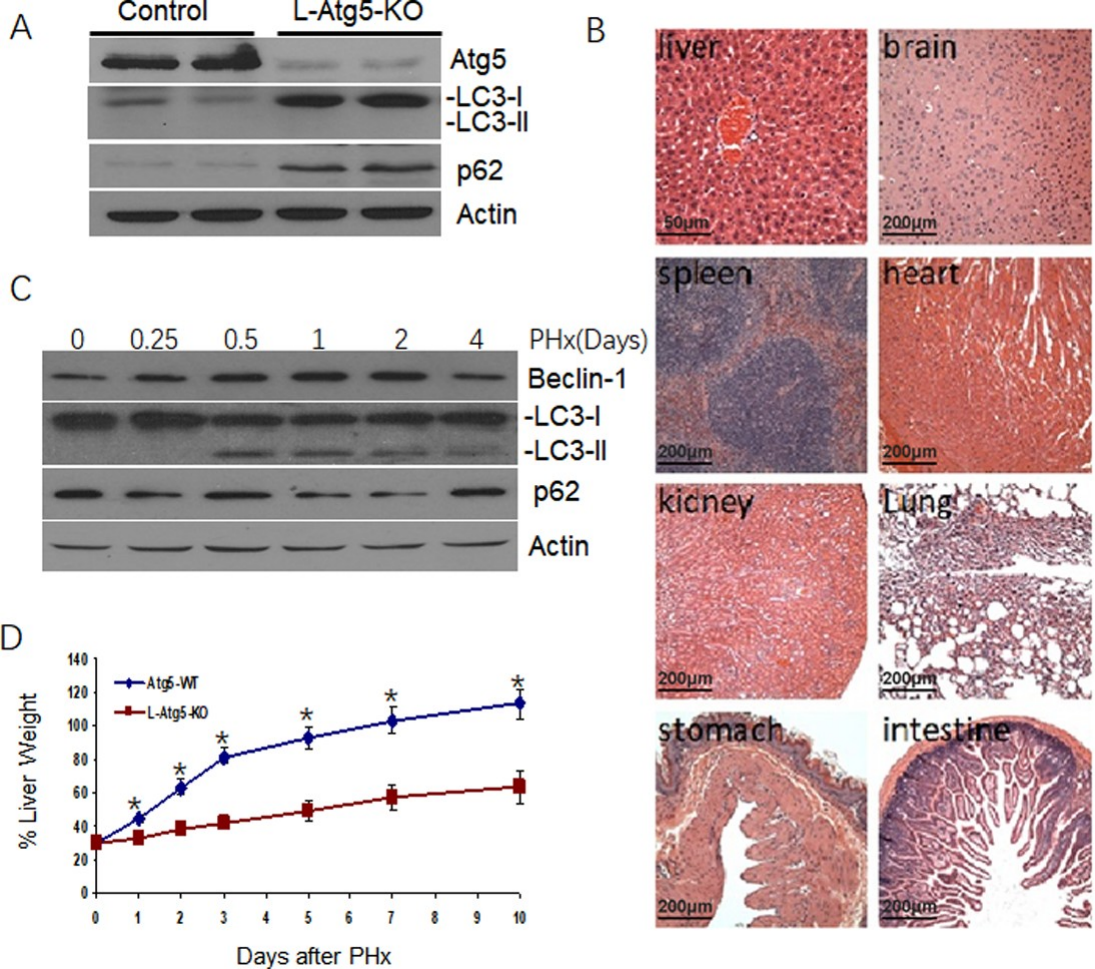
Development of hepatic tumors in L-Atg5-KO mice. (A) Immunoblot analysis of Atg5, LC3 and p62 in the liver of 10-weeks old Atg5-WT and L-Atg5-KO mice. Actin served as the loading control. LC3-I, non-lipidated LC3; LC3-II, lipidated LC3. Two or more tissue samples were analyzed to ensure the reproducibility of the results. (B) Hematoxylin and eosin staining of the liver tissue sections of Atg5-WT and L-Atg5-KO mice. (C) Autophagic activation in the regenerating liver after PHx. Immunoblot analysis of Beclin-1, LC3 and p62 in the liver at 6 hours, 0.5 daysy, 1 day, 2 days and 4 days after PHx in control mice. Actin served as the loading control. (D) Liver regeneration rates of Atg5-WT and L-Atg5-KO mice. Ten-weeks old Atg5-WT and L-Atg5-KO mice were subjected to 70% PHx and sacrificed at various time points after the surgery for liver weight measurements. Five mice were analyzed per time point and the results represented the mean. The average liver weight prior to PHx was defined as 100%. The results represent the mean ± s.e.m. of at least three mice. *, *p*<0.05; **, *p*<0.01.

We generated C57BL/6 mice with liver-specific knockout of *Atg*5 (L-Atg5-KO). Immunoblot analysis showed that only few Atg5 was detected in the liver of the L-Atg5-KO mice, indicating a successful knockout of this gene (Fig 1A). Atg5 is depleted only in liver, not in other organs and shown the results in S1 Fig. The deletion of the *Atg5* gene inhibited the lipidation of LC3 and increased the non-lipidated LC3 protein level in the liver. An increase of the p62 protein level was also observed. As the lipidation of LC3 is essential for the formation of autophagosomes and p62 is a protein removed by autophagy [22], these results confirmed that the liver-specific knockout of Atg5 impaired autophagy in the mouse liver. Histological analysis of liver tissue sections of the mice liver and other organs, including the brain, heart, kidney, stomach, intestine and spleen were normal (Fig 1B).

### Reduction of hepatocellular proliferation in L-Atg5-KO mice

To investigate the possible effect of autophagy on liver regeneration, we firstly analyze the effect of PHx on liver autophagy in control mice. 70% PHx caused an increase of the beclin-1 and LC3-II, peaking at 0.5 days after PHx, which was maintained until 2 days, and returned to baseline after 4 days. Expression of p62 was reduced from 1 day to 2 days after PHx and returned to normal level at 4 days after PHx (Fig 1C). It indicated that autophagy was activated during liver regeneration induced by PHx. The liver of control mice underwent rapid regeneration and reached approximately 100% of the original liver weight ten days after the surgery (Fig 1D). We analyzed the hepatocellular proliferation rate in L-Atg5-KO mice after 70% PHx. As shown in Fig 1D, the liver regeneration rate of L-Atg5-KO mice was significantly lower (*p*<0.05) at every time point after PHx and regained only about half of the original liver weight ten days after the surgery. It demonstrated that liver regeneration in the L-Atg5-KO mice after PHx was impaired.

### No apparent difference of hepatic cytokines response between WT and L-Atg5-KO mice after PHx

**Fig 2:**
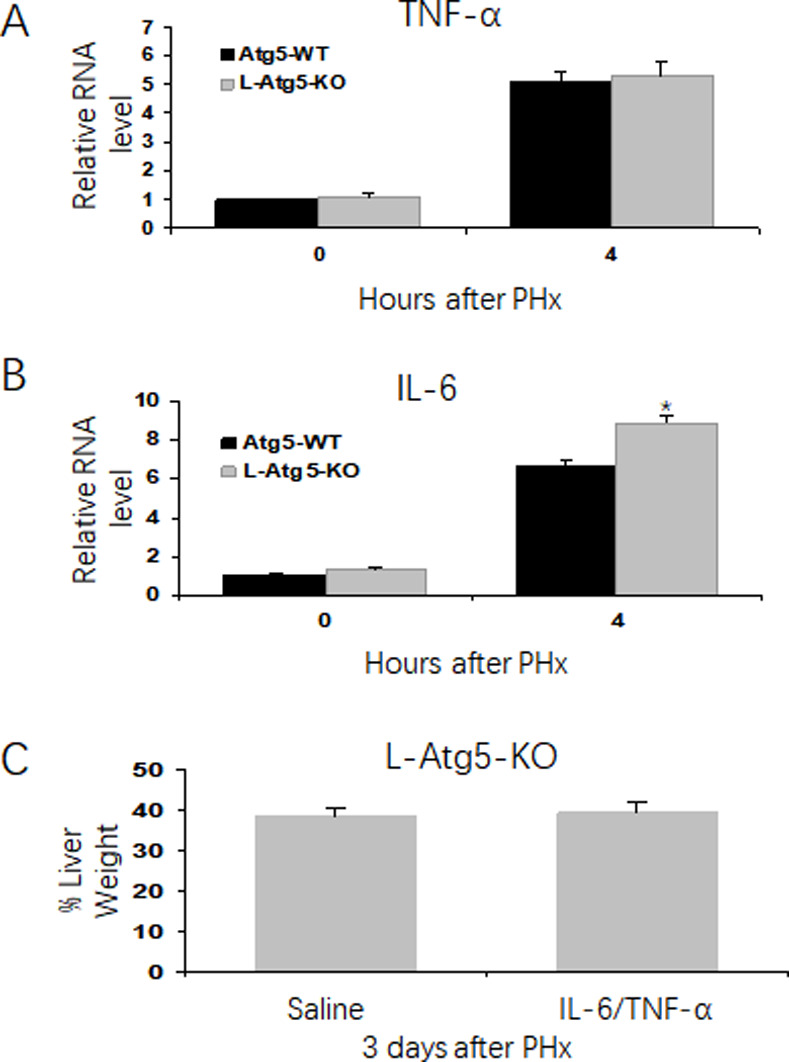
Impaired hepatic liver proliferation is not associated with cytokines response in the regenerating liver in control and L-Atg5 KO mice. Analysis of IL-6 (A) and TNF-α (B) mRNA levels in the mouse liver tissue by real-time RT-PCR. The RNA level of wild-type mice was arbitrarily defined as one. (C) L-Atg5-KO mice were injected with IL-6 and TNF-α (500 μg/kg), or saline at two hours before PHx and one day after PHx. The mice were sacrificed at 3 days after PHx, and the liver regeneration rate was measured (Three mice for each group). The average liver weight prior to PHx was defined as 100%. *, *p*<0.05, Data were analyzed by the Student’s *t*-test.

To investigate why liver regeneration was impaired in L-Atg5-KO mice, we analyzed the expression of IL-6 and TNF-α in the liver of both control and L-Atg5-KO mice after PHx, as IL-6 and TNF-α are two key cytokines important for the initiation of liver regeneration. As shown in Fig 2A and 2B, the mRNA levels of TNF-α and IL-6 in the liver significantly increased in both control and L-Atg5-KO mice at 4 hours after PHx. There was no significant difference between the TNF-α mRNA levels of WT and L-Atg5-KO mice at 4 hours after PHx (Fig 2A), and the IL-6 mRNA level was actually slightly higher in L-Atg5-KO mice than in control mice at this time point (Fig 2B). These results indicated that the ablation of autophagy in hepatocytes did not negatively affect the expression of TNF-α and IL-6 immediately after PHx. To confirm that TNF-α and IL-6 were indeed not the reason why the liver regeneration in L-Atg5-KO mice was impaired, we treated the L-Atg5-KO mice with IL-6 and TNF-α two hours before and one day after PHx. As shown in Fig 2C, this treatment did not increase the liver mass of L-Atg5-KO mice 3 days after PHx, confirming that these two cytokines were not the reason why the liver regeneration of L-Atg5-KO mice was impaired.

### Impeded and prolonged hepatocellular proliferation in L-Atg5-KO mice after PHx

**Fig 3:**
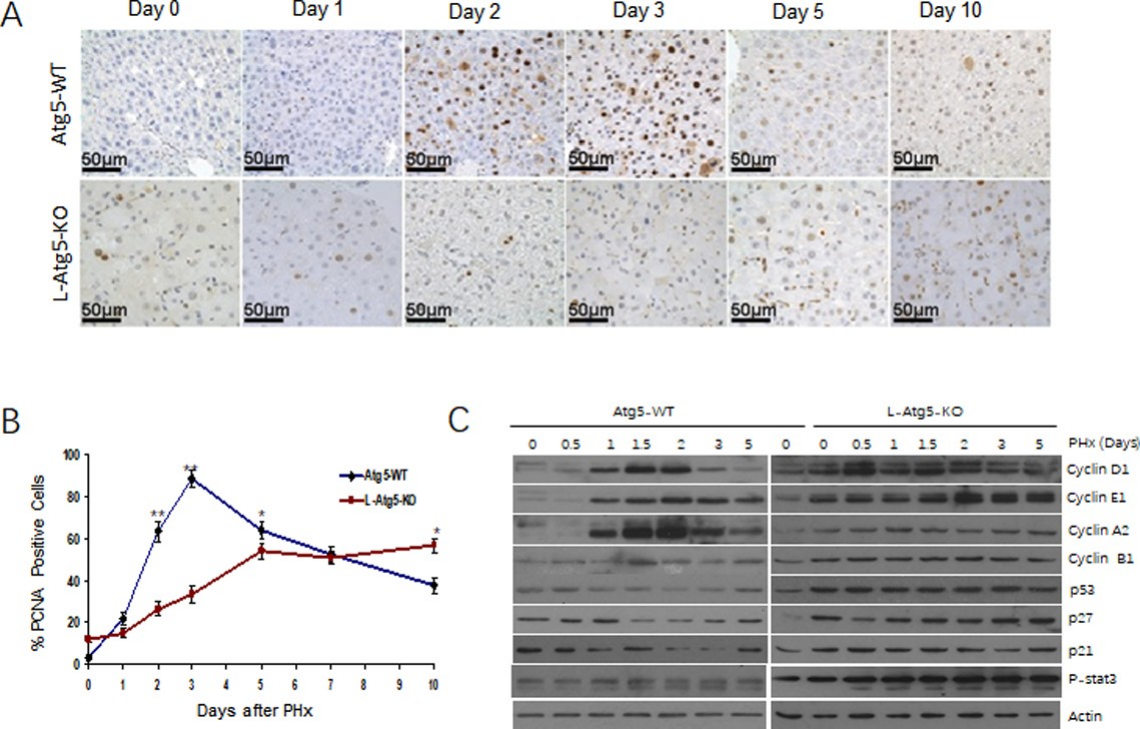
DNA synthesis during liver regeneration is attenuated and delayed in L-Atg5-KO mouse livers. (A) Immunostaining of PCNA at indicated time points after PHx. (B) Percentage of PCNA-positive cells after PHx. The results represented the mean of 20 different viewing fields. The differences between wild-type and L-Atg5-KO mice from day 0 to day 5 and on day 10 were statistically significant (*p*<0.05). (C) Immunoblot analysis of changes in cyclins D1, E1, A2, B1, p53, p27, as well as p21 protein expression levels in Atg5-WT and L-Atg5 KO mice at different time points after 70% PHx. Actin was used as a loading control.

To further investigate how the defect in autophagy impaired the liver regeneration, we compared the hepatocellular proliferation rate between control and L-Atg5-KO mice by staining liver tissue sections for PCNA, a nuclear protein associated with cellular proliferation, at different time points after PHx. As shown in Fig 3A and 3B, there were few PCNA-positive cells in the control mouse liver before PHx. However, the number of PCNA-positive cells increased to about 50% of total hepatocytes on day 2 and nearly 70% on day 3. The percentage of PCNA-positive cells declined thereafter. In contrast, about 10% of hepatocytes of L-Atg5-KO mice were PCNA-positive even before PHx, possibly due to hepatocellular apoptosis and the ensuing liver regeneration (see below). This number increased slowly after PHx, reached about 50% on day 5 and remained roughly at that level on day 10. These results indicated that the ablation of autophagy by Atg5 knockout impeded and prolonged hepatocellular proliferation after PHx.

### Dysregulated expression of cyclins and tumor suppressors in the L-Atg5-KO mouse liver

To understand why hepatocellular proliferation was impeded and prolonged in L-Atg5-KO mice, we analyzed the expression of cyclins D1, E1, A2 and B1in the liver of control and L-Atg5-KO mice at different time points after PHx. These cyclins regulate the progression and transition of different phases of the cell cycle via the regulation of the activities of cyclin-dependent kinases. As shown in Fig 3C, the expression of cyclins D1, E1 and A2, which regulate the G1 phase progression, the G1/S phase transition and the S phase progression, respectively [23–26], was induced at 1 day after PHx in the WT mouse liver. Their induction peaked at 2 days and declined thereafter. The induction of cyclin B1, which regulates the G2/M phase transition [27], was also observed at 1.5 days after PHx. These results were consistent with the results shown in Fig 3A and 3B, which revealed a high degree of hepatocellular proliferation at 2 and 3 days after PHx. The expression profiles of cyclins in the liver of L-Atg5-KO mice, however, were different from those of control mice. As shown in Fig 3C, the expression levels of cyclins D1, E1, A2 and B1 were all higher in the liver of L-Atg5-KO mice than in control mice before PHx. A slight increase was observed for cyclins D1 and A2 during 0.5–1.5 days after PHx and for cyclin E1 1.5 days after PHx. No increase of cylcin B1 was observed after PHx. When the tumor suppressors p53, p21 and p27 in the control mice liver were analyzed, their expression levels were found to be transiently decreased during 1–3 days for p53 and p21, and 1.5–3 days for p27. These results were expected, as these tumor suppressors are known to inhibit cell proliferation. In contrast, the expression levels of p53, p27 and p21 were all higher in L-Atg5-KO mice than in WT mice prior to PHx. The expression of these tumor suppressors was not reduced nor further increased in the liver of L-Atg5-KO mice after PHx. The observations of dysregulated expression of cyclins and increased expression of tumor suppressors provided an explanation to why the hepatocellular proliferation in L-Atg5-KO mice after PHx was impaired.

### Induction of the intrinsic apoptotic pathway in liver of L-Atg5-KO mice

**Fig 4:**
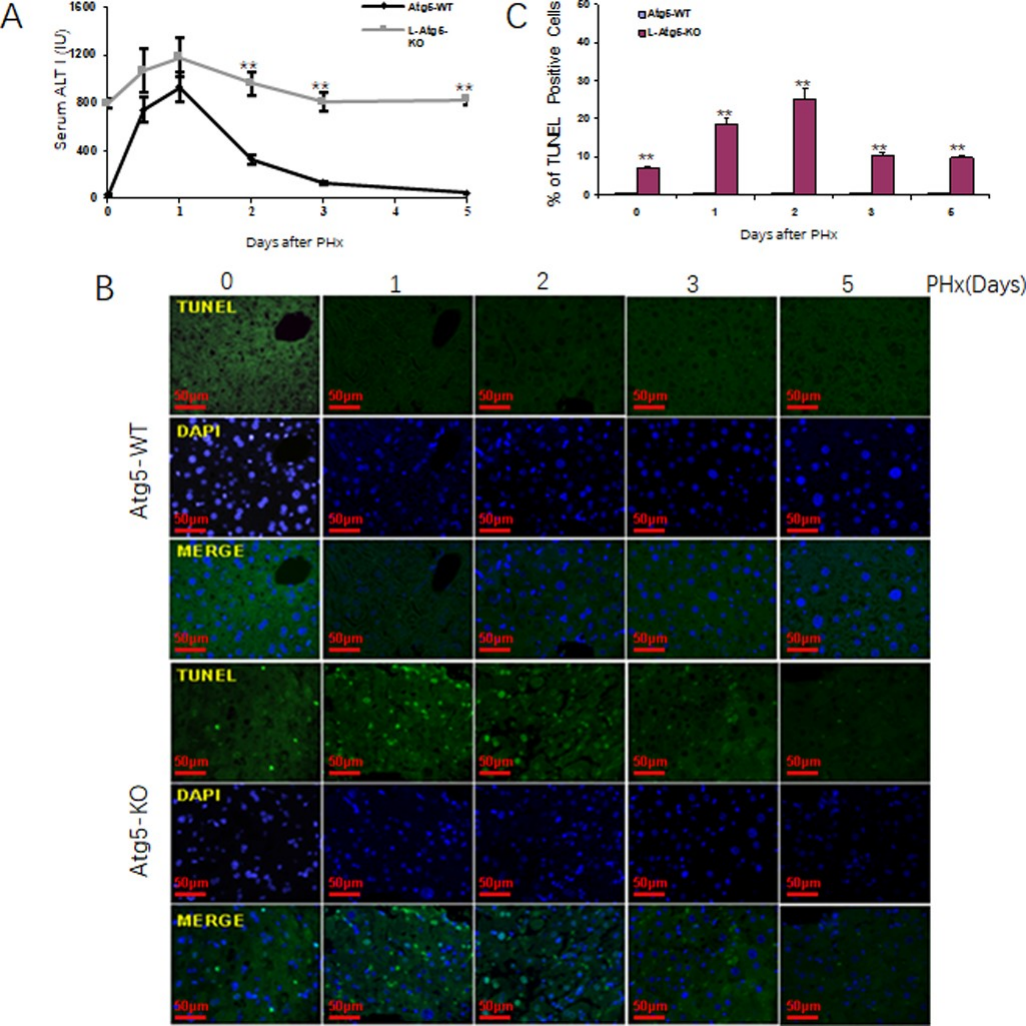
Induction of apoptotic in the liver of L-Atg5-KO mice after PHx. (A) Serum ALT level was measured at indicated time pointed. Five mice were analyzed per time point and the results represented the mean. (B) TUNEL staining in liver tissue section of L-Atg5-KO mice at different time points after PHx. (C) Percentage of TUNEL-positive cells after PHx. The results represented the mean of 20 different viewing fields statistically significant. The results represent the mean ± s.e.m. of at least three mice. *, *p*<0.05; **, *p*<0.01.

The observation that approximately 10% of hepatocytes in L-Atg5-KO mice were PCNA-positive prompted us to examine whether these PCNA-positive hepatocytes were the result of compensatory cell proliferation caused by a low level of cell deaths in L-Atg5-KO mice. We first measured the alanine aminotransferase (ALT) levels in the mouse serum. As shown in Fig 4A, a high ALT level was indeed observed in L-Atg5-KO mice, indicative of liver injury. This ALT level was increased by PHx and peaked on day 1 after PHx. In contrast, the ALT level in control mice was very low before PHx and increased significantly within the first 24 hours after PHx. This level dropped after 1 day and returned to the basal level on day 5 after PHx. To understand the mechanism of liver injury in L-Atg5-KO mice, we conducted the TUNEL assay to analyze apoptotic cells in liver tissue sections prepared from control and L-Atg5-KO mice. As shown in Fig 4B, although few TUNEL-positive cells could be detected in control mice liver tissues before and after PHx, approximately 9% of hepatocytes were TUNEL-positive in L-Atg5-KO mice before PHx. The percentage of TUNEL-positive hepatocytes in L-Atg5-KO mice were further increased by PHx and reached ~18% on day 2 after PHx (Fig 4C). This percentage was reduced thereafter. The induction of hepatocellular apoptosis by PHx provided another explanation to why liver regeneration was impaired in L-Atg5-KO mice.

**Fig 5:**
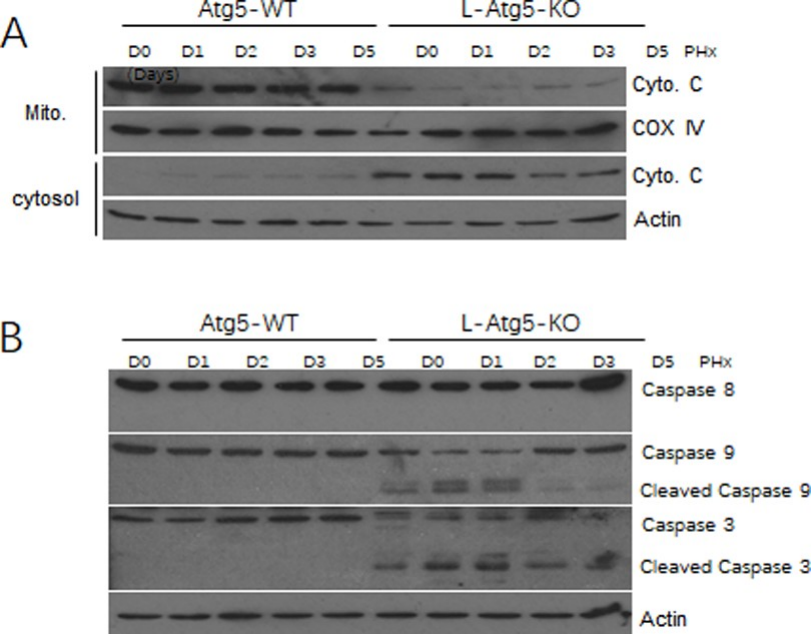
The intrinsic apoptotic pathway was activated in the liver of L-Atg5-KO mice after PHx. (A) Immunoblot analysis of caspases, cytochrome C, which is a mitochondria membrane protein [31], COX IV was used as a loading control for the mitochondrial fraction (Mito). Actin was used as the loading control for the cytosolic fraction. (B) Immunoblot analysis of caspase-8, caspase-9 and caspase-3 levels in the liver tissue of Atg5-WT and L-Atg5-KO mice. Actin was used as a loading control.

To investigate the mechanism of hepatic apoptosis in L-Atg5-KO mice, we conducted the subcellular fractionation experiments of liver tissues to separate mitochondria and the cytosol and analyzed the level of cytochrome C in these two fractions. Cytochrome C is released from mitochondria under stress conditions and its release can activate the intrinsic apoptotic pathway, which is characterized by the cleavage of procaspases 9 and 3, but not procaspase 8, which is cleaved when the extrinsic apoptotic pathway mediated by death receptors is activated [28–30]. As shown in Fig 5A, the cytochrome C level in the mitochondrial fraction was significantly lower in L-Atg5-KO mice than in WT mice before and after PHx at every time point, particularly on day 1 and day 2 after PHx. This reduction of cytochrome C in the mitochondrial fraction of L-Atg5-KO mice was associated with a concomitant increase of cytochrome C in the cytosolic fraction, with the increase being most prominent on day 1 and day 2 after PHx. We had also analyzed the possible cleavage of procaspases 8, 9 and 3 by immunoblot (Fig 5B). Actin in mitochondria and COX IV in cytoplasm were blotted successful in isolation. The results shown that there was no actin visible in isolated mitochondria, and there was no COX IV visible in isolated cytoplasm as shown in S2 Fig. They are stained in the same batch with exactly the same exposure time for each gene. Although no cleavage of any of these procaspases was observed in the wild-type mouse liver, the cleavage of procaspases 9 and 3, but not procaspase 8, was observed in the liver tissues of L-Atg5-KO mice both before and after PHx, again with the cleavage being most prominent on day 1 and day 2 after PHx. These results indicated that the apoptosis of hepatocytes in L-Atg5-KO mice was due to the specific activation of the intrinsic apoptotic pathway and this activation was enhanced during the first two days after PHx.

### Altered metabolism during liver regeneration in L-Atg5-KO mice

**Fig 6:**
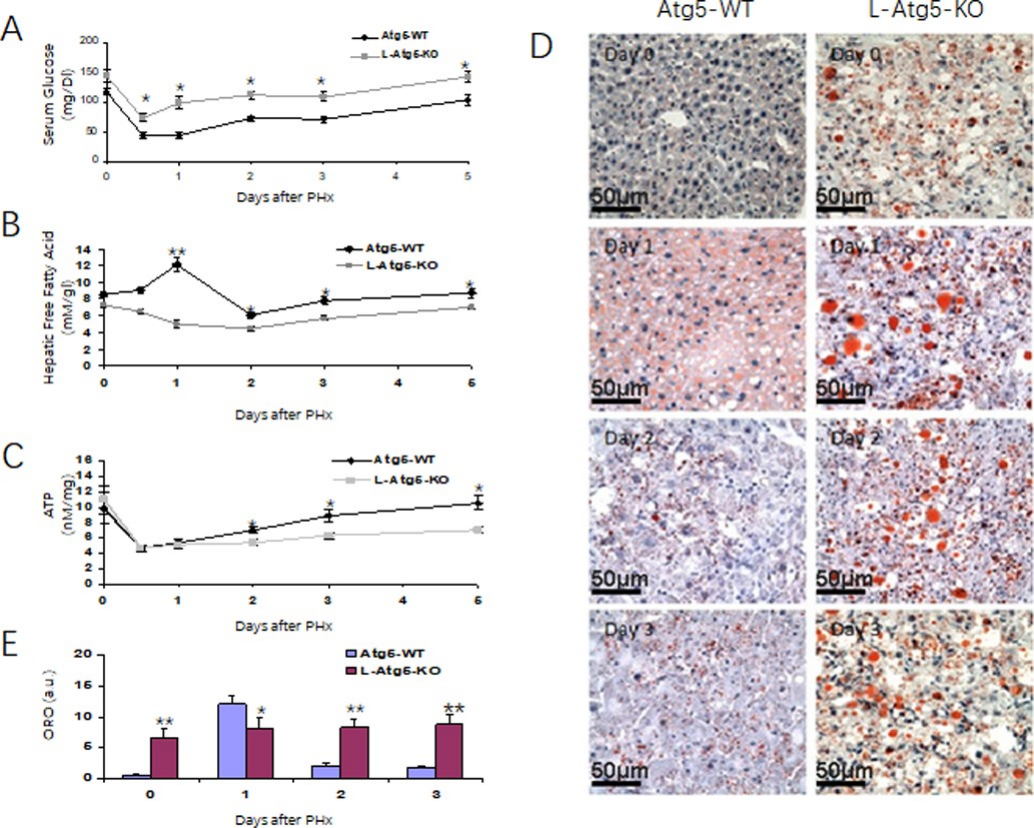
The metabolic responses to PHx in Atg5-WT and L-Atg5-KO mice. Serum glucose levels (A), Hepatic free fatty acid levels (B) and Hepatic ATP levels (C) were measured at serial times after PHx. (D) Neutral lipid accumulation in liver tissues from PHx mice was analyzed by oil red O staining. (E) Quantification of neutral lipids at indicated time points. The results represented the mean of 20 different viewing fields statistically significant. In (A), (B) and (C), 5 mice were analyzed for every time point and the results represented the mean ± s.e.m. of at least three mice. *, *p*<0.05; **, *p*<0.01.

PHx could induce hypoglycemia and this reduction of blood glucose had been shown to be important for liver regeneration, which could be suppressed by glucose supplementation and accelerated by dietary caloric restriction [32–34]. For that reason, we analyzed the possible difference of the blood glucose levels between WT and L-Atg5-KO mice. As shown in Fig 6A, the serum glucose level was higher in L-Atg5-KO mice than in WT mice before and at every time point after PHx, indicating a possible role of this increased serum glucose level on the suppression of liver regeneration.

Similarly, previously studies indicated that lipids could be an advantageous energy source for regenerating liver and impairing fat metabolism had been shown to inhibit liver regeneration [34]. For that reason, we also investigated whether the reduced liver regeneration in L-Atg5-KO mice was also associated with the disorder of lipid metabolism by measuring the free fatty acid (FFA) level in the liver. As shown in Fig 6B, the hepatic FFA level of WT mice increased significantly one day after PHx. It then declined on day 2 and returned to the basal level on day 5. In contrast, the hepatic FFA level of L-Atg5-KO mice decreased after PHx and reached the lowest level on day 2. It then returned to the original level on day 5 after PHx.

FFA via β-oxidation provides the predominant source of ATP during liver regeneration [32–34]. Its reduction in the liver of L-Atg5-KO mice would thus likely affect the hepatic ATP levels. To test this possibility, we also measured the hepatic ATP levels. As shown in Fig 6C, there was little difference between the hepatic ATP levels of WT and L-Atg5-KO mice before PHx and a similar reduction of ATP levels in these two groups of mice during the first 24 hours after PHx. However, while the ATP level of WT mice was restored to the original level 120 hours after the surgery, the restoration of the ATP level in L-Atg5-KO mice was inefficient and reached only 60% of the original level at 120 hours after PHx. Thus, these reductions of hepatic FFA and ATP levels likely also contributed to the reduction of liver regeneration in L-Atg5-KO mice.

To further investigate why the FFA level decreased and not increase in L-Atg5-KO mice after PHx, we stained liver tissue sections for lipid droplets with oil red. As show in Fig 6D, an increased level of lipid droplets was observed in the hepatocytes of WT mice one day after PHx. This level was reduced on day 2 and day 3 after PHx (Fig 6E). In agreement with the previous report [35], the number of lipid droplets in the hepatocytes of L-Atg5-KO mice were significantly higher than those in the hepatocytes of WT mice before PHx. The size and the quantity of lipid droplets were further increased in L-Atg5-KO mice on day 1 after PHx with no significant reduction on day 2 and day 3. These results indicated that inability of PHx to increase hepatic FFA in L-Atg5-KO mice was likely due to a defect in lipolysis in mouse hepatocytes.

## Discussion

In this report, by using PHx to induce liver regeneration, we demonstrated an important role of autophagy in facilitating liver regeneration. We found that the ablation of autophagy could impede the process of liver regeneration (Fig 1D), and this impairment of liver regeneration was not due to the lack of expression of IL-6 and TNF-α, which play an important role in the initiation of liver regeneration after liver injury (Fig 2). This result was not surprising, as TNF-α and IL-6 are mainly produced by hepatic macrophages (i.e., Kupffer cells) [36], and the ablation of autophagy in hepatocytes would not be expected to affect the production of these two cytokines by Kupffer cells. Instead, we found that this impairment of liver regeneration was due to the reduction of hepatocellular proliferation rate (Fig 3A and 3B), which was associated with the dysregulated expression of cyclins and increased expression of tumor suppressors (Fig 3C).

We also found that the ablation of autophagy could activate the intrinsic apoptotic pathway to cause hepatocellular deaths, which were exacerbated by PHx (Figs 4 and 5). Thus, the ablation of autophagy could reduce the hepatocellular proliferation rate and at the same time increase the hepatocellular death rate. Their combined effects led to the reduction of liver regeneration rate and prolonged the liver regeneration process.

In response to liver injury, there are rapid and specific hepatic and systemic metabolic alterations [20]. Indeed, we were able to observe a rapid but transient reduction of the serum glucose level, a transient increase of the hepatic FFA level, a transient reduction of the hepatic ATP level in WT mice (Fig 6A–6C). This was coupled with a transient development of steatosis in the regenerating liver, which peaked at 24 hours after PHx (Fig 6D). The transient steatosis was likely the result of hypoglycemia, which could increase the hepatocellular uptake of adipose-derived fat stores, as previously suggested [20]. Similarly, the transient increase of hepatic FFA was likely also due to the lipolysis of adipose. These alterations are important for liver regeneration, as glucose supplementation suppressed liver regeneration [32–34], and both dietary and parenteral administration of lipid-based formulations accelerated it [32–34]. In addition, FFA via β-oxidation serves as the predominant source of new ATP production to meet intrahepatic energy demands [34, 37]. In contrast to WT mice, there was no transient increase of hepatic FFA in L-Atg5-KO mice. As steatosis increased and persisted in these mice (Fig 6D), it is conceivable that the deficiency in autophagy, which plays an important role in regulating lipid metabolism [35, 38], led to inefficient lipolysis and the reduction of hepatic FFA. This reduction of hepatic FFA in turn led to the inefficient restoration of the hepatic ATP level in these mice. The reason why L-Atg5-KO mice had elevated levels of blood glucose during liver regeneration, as compared with the WT mice, is unclear. It is interesting to note that the blood glucose level of L-Atg5-KO mice prior to PHx was also higher than that in WT mice, suggesting a possible defect in gluconeogenesis. In any case, the decrease of hepatic FFA and ATP levels as well as the increase of blood glucose level in L-Atg5-KO mice during liver regeneration likely also played a critical role in impeding liver regeneration L-Atg5-KO mice.

Autophagy plays an important role in liver function, physiology and pathology. Many functions of liver also depend on autophagy of liver cells. First of all, the liver has a unique regeneration ability. In liver regeneration, liver cells eliminate cells that cannot be renewed and metabolites through autophagy. When damaged organelles, oxidation products and assembled proteins cannot be degraded through autophagy timely, various liver diseases will be generated. Secondly, autophagy has the function of regulating liver metabolism and liver cell proliferation. The products of autophagy, such as amino acids and fatty acids, can not only maintain the liver’s own capacity, but also synthesize glucose and store fat to provide energy for the whole body. Finally, the liver has a unique anatomical structure, and the portal vein receives blood directly from the intestines. The liver may resist foreign antigens, metabolites of intestinal bacteria such as lipopolysaccharides and viruses through autophagy, which plays an important role in the immune regulation of the liver.

Mitochondria are rich in proteins and lipids, and about 85% hepatocytes select mitochondrial autophagy when lack of nutrients [39, 40]. Hepatocytes remove damaged and aging mitochondria through autophagy, prevent the release of pro-apoptotic proteins, inhibit the production of toxic reactive oxygen species (ROS) and mitochondrial depolarization, and play a certain protective role on liver cells. After knockout Atg7 in mice with normal liver in 90 days, the liver weight increased nearly four times, and histological study shown lobular architecture disappear at this time, liver cell swelling, filled with a contain mitochondria vacuoles and lipid droplets, abnormal peroxidase in liver cells, mitochondria membrane structure and the increase in the number, eventually cause liver cell death [41]. The triglyceride and cholesterol in mouse liver were significantly increased after Atg7 gene was knockout. Therefore, autophagy-mediated fat metabolism mechanism plays an important role in regulating intracellular lipid storage and fat consumption [35].

Autophagy plays an important regulatory role in liver regeneration. Early stress signals in liver regeneration may be the result of increased energy requirements per unit of increased liver mass. After liver resection, the remaining liver tissue retains liver specific functions such as gluconeogenesis, urea synthesis and ATP synthesis [42, 43]. The inhibition of autophagy leads to the accumulation of SQSTM1/ p62, which acts as a signaling to activate the NF-κB signaling pathway, promoting hepatocyte proliferation and activating transcription of genes encoding antioxidant proteins and detoxification enzymes through Nrf2 transcription factors [44]. Subsequently, due to the large demand of cells for energy, autophagy degrades intracellular proteins, nucleic acids and other cellular components to provide ATP for hepatocyte division with the recombination of intracellular proteins and organelles, then it promotes the division and proliferation of hepatocytes [45].

Autophagy is conducive to the metabolism and breakdown of nutrients, which is crucial to the regulation of nutrients and maintenance of intracellular balance. Biomolecules can be degraded to glucose, free fatty acids and amino acids by autophagy lysosome pathway for the reuse and energy of cell. When glucose and lipids are consumed in abundance, autophagy occurs to degrade the cell’s energy stores. In the absence of nutrients, autophagy can be used to mobilize glycogen and fat in cells to generate energy. Autophagy can also promote the degradation of proteins in cells to provide amino acids to maintain protein synthesis [46, 47].

There is a complex interaction between autophagy and apoptosis, which can be activated jointly by multiple stress stimuli, share multiple regulatory molecules, and even coordinate their transformation. A comprehensive and in-depth study of the interaction mechanism between autophagy and apoptosis will bring breakthroughs in the cognition and treatment of liver diseases and other diseases. Autophagy plays an important role in bulk degradation of cytoplasm and mitochondrion. Autophagy influences mitochondrial recycle and can thus modulate hepatic apoptosis via mitochondrial pathway. Inflammasome dependent caspase-1 activation promotes pyroptotic cell death and the secretion of proinflammatory cytokines. Autophagosome formation depends on the covalent binding of a series of Atg proteins during protein ubiquitination. Recent advances have confirmed the interaction between autophagy and apoptosis, which are manifested by regulatory genes that are shared with common pathways, including p53, Atg5, Bcl-2, and so on. The autophagic protein Atg5 contributes to autophagic cell death well known in autophagy. Atg5 can stimulate extrinsic apoptosis pathways and induces apoptotic death that can be blocked by pan caspase inhibitor Z-VAD-fmk by interacting with fas-associated protein with death domain [48]. Atg5 participates in the formation of autophagosomes and enhances the sensitivity to apoptotic stimuli as well. calpain-mediated Atg5 cleavage was associated with apoptosis, which is independent of the cell type and the apoptotic stimulus. The generation of truncated Atg5 is dependent by calpain and then translocate from the cytosol to mitochondria, which inhibits the anti-apoptotic Bcl-xL and provokes cytochrome C release and caspase activation. Atg5 and Atg12 are known as the "core" of autophagy and are necessary for autophagosome formation. They have also been found to be involved in the regulation of apoptosis. Atg5 could be cut by calpains, resulting in the translocation of the N-terminal fragment of Atg5 to mitochondria by an unknown mechanism, which combined with the anti-apoptotic protein Bcl-XL to promote the release of mitochondrial cytochrome C and induce apoptosis [49]. TNF-α-dependent activation of proapoptotic caspases are prevented by the knockout of Atg5 [50]. The deletion of Atg5 could protect cells from pro-death environmental stimuli and this resistance may result from compensatory activation of chaperone mediated autophagy, rather than inhibition of macroautophagy [51]. Recent studies have elucidated the mechanisms of liver regeneration including cytokines, growth factors, and metabolic networks [36]. Hepatocytes progress through the cell cycle in response to a collection of mitogenic growth factors. Liver progenitor cells as the second line defense against liver injury, becoming active when mature hepatocytes are prevented from proliferating [52]. It has been postulated that at 50% loss of hepatocytes with decreased proliferation of mature hepatocytes triggers proliferation of the progenitor cell population [53]. Endocannabinoids acting via cannabinoid type 1 receptors (CB1R) promote neural progenitor cell proliferation, and they promote lipogenesis in the liver. These findings suggest CB1R involve in the control of liver regeneration. Activation of hepatic CB1R by newly synthesized anandamide promotes liver regeneration by controlling the expression of cell-cycle regulators that drive M phase progression [54]. As a pivotal mediator of acute and chronic liver injury, the endocannabinoid system has emerged with the description of the role of CB1 and CB2 receptors and their endogenous lipidic ligands in various aspects of liver pathophysiology [55].

In conclusion, our study demonstrated a critical role of autophagy in liver regeneration. When autophagy is defective, hepatocellular proliferation is impaired, hepatocellular apoptosis is induced, and hepatic and systemic metabolisms are altered, resulting in impaired liver regeneration after liver injury.

## Supporting information

S1 Fig Liver-specific of Atg5 knockout. (A) Immunoblot analysis of changes in Beclin-1, LC-I and LC-II protein expression levels in Atg5-WT in the different organs. Actin was used as a loading control. (B) Immunoblot analysis of changes in Beclin-1, LC-I and LC-II protein expression levels in L-Atg5 KO mice in the different organs.
(TIF)

S2 Fig Actin in mitochondria and COX IV in cytoplasm were isolated successfully. (TIF)

S1 Raw images (PDF)

## List of abbreviations

PHx: partial hepatectomy
ALT: alanine aminotransferase
Atg: autophagyrelated gene
ATP: adenosine 50-triphosphate
IHC: immunohistochemistry
IL: interleukin
KO: knockout
LC3: microtubule-associated protein 1 light chain 3
LW/BW: liver weight/body weight ratio
mRNA: messenger RNA
NF-κB: nuclear factor kappa B
PCR: polymerase chain reaction
PHx: partial hepatectomy
PCNA: proliferating cell nuclear antigen
TNF: tumor necrosis factor
IL-6: interleukin-6
NF-κB: nuclear factor kappa B
STAT3: signal transducer and activator of transcription 3
TNFR1: type I TNF-receptor
TGF-α: transforming growth factor alpha
EGF: epidermal growth factor
HGF: hepatocyte growth factor
LPS: lipopolysaccharide
TLR: Toll-like receptor
cdk: cyclin-dependent kinase
EGFR: EGF receptor
