# Supplementary figures and images for: Altered metabolism by autophagy defection affect liver regeneration
Source: PLoS One. 2021 Apr 29;16(4):e0250578. doi: 10.1371/journal.pone.0250578 (PMC8084245; doi:10.1371/journal.pone.0250578)

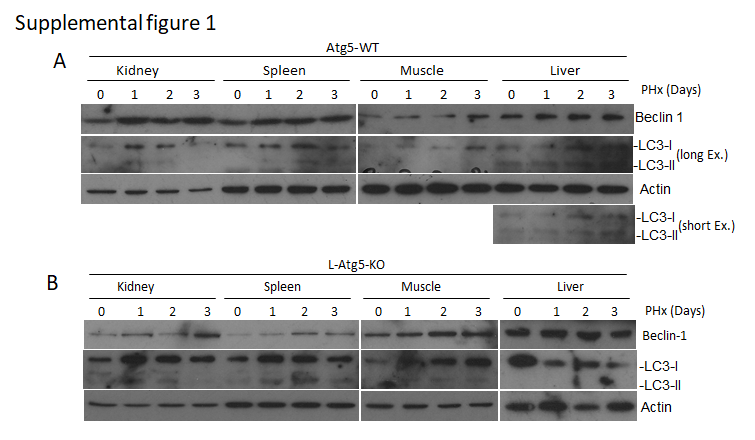

Supplement: S1 Fig — (A) Immunoblot analysis of changes in Beclin-1, LC-I and LC-II protein expression levels in Atg5-WT in the different organs. Actin was used as a loading control. (B) Immunoblot analysis of changes in Beclin-1, LC-I and LC-II protein expression levels in L-Atg5 KO mice in the different organs. (TIF) [file pone.0250578.s001.tif]

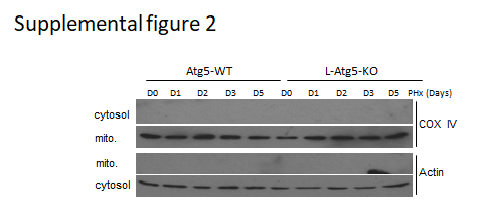

Supplement: S2 Fig — (TIF) [file pone.0250578.s002.tif]
