# Supplementary material for: Altered metabolism by autophagy defection affect liver regeneration
Source: PLoS One. 2021 Apr 29;16(4):e0250578. doi: 10.1371/journal.pone.0250578 (PMC8084245; doi:10.1371/journal.pone.0250578)
Supplement: S1 Raw images — (PDF) [file pone.0250578.s003.pdf]

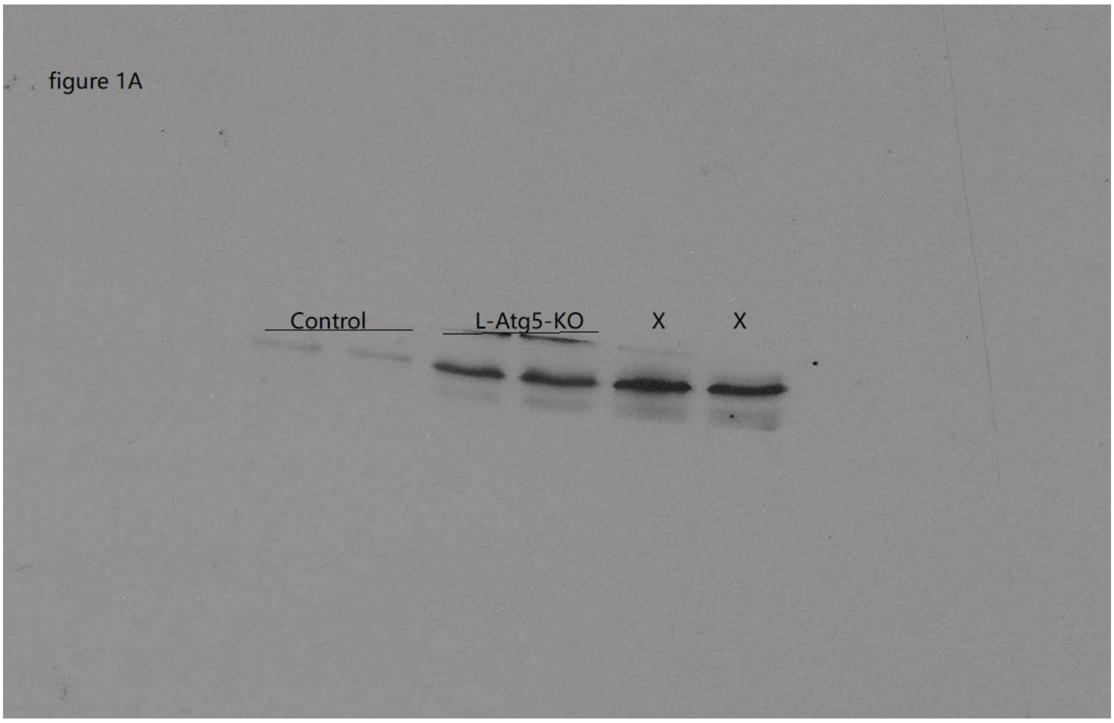
figure 1A
Control
L-Atg5-KO
X
X

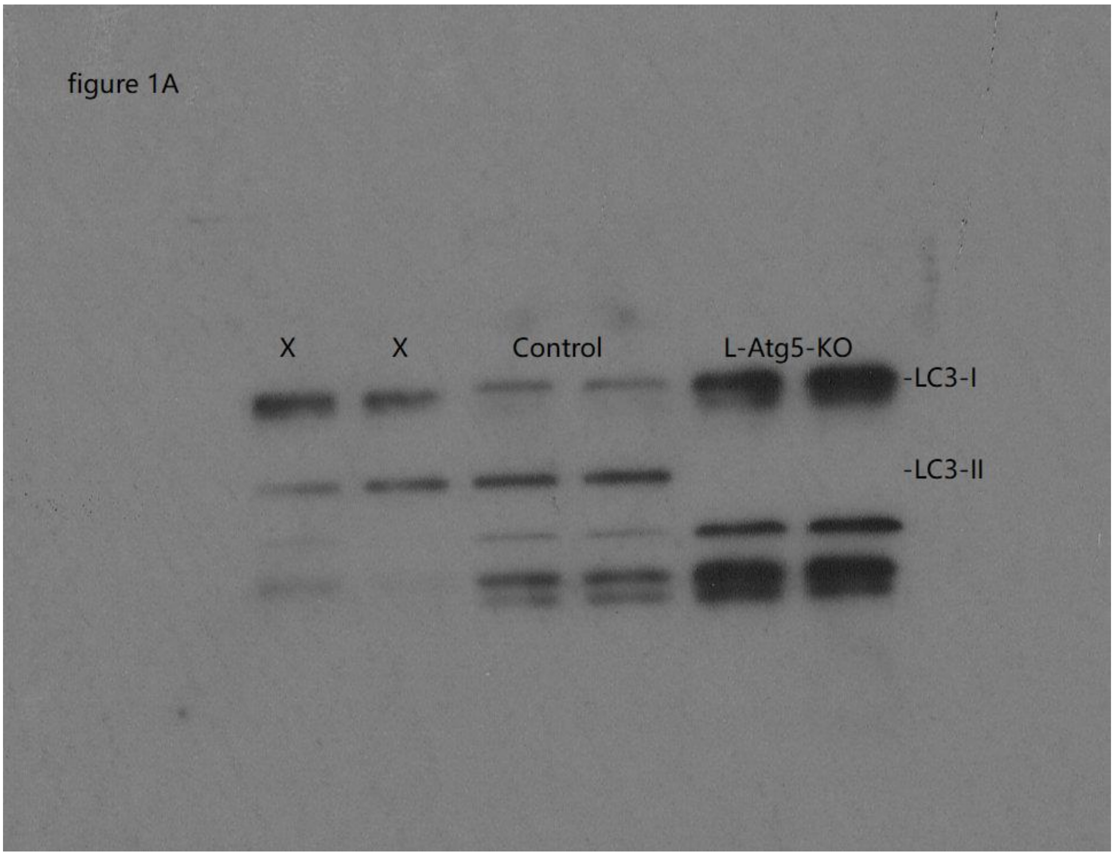
figure 1A
X
X
Control
L-Atg5-KO
-LC3-I
-LC3-II

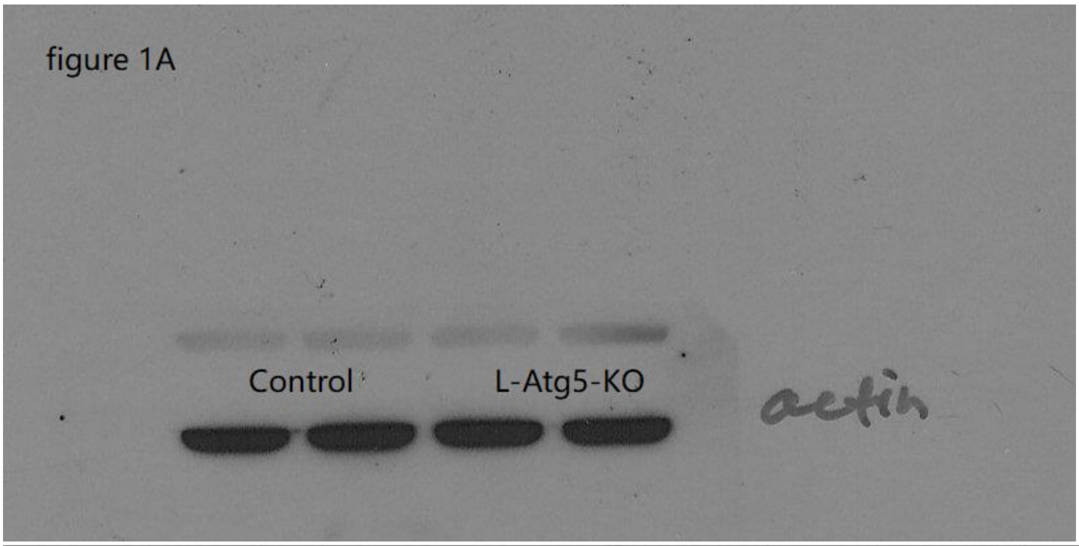
figure 1A
Control
L-Atg5-KO
actin

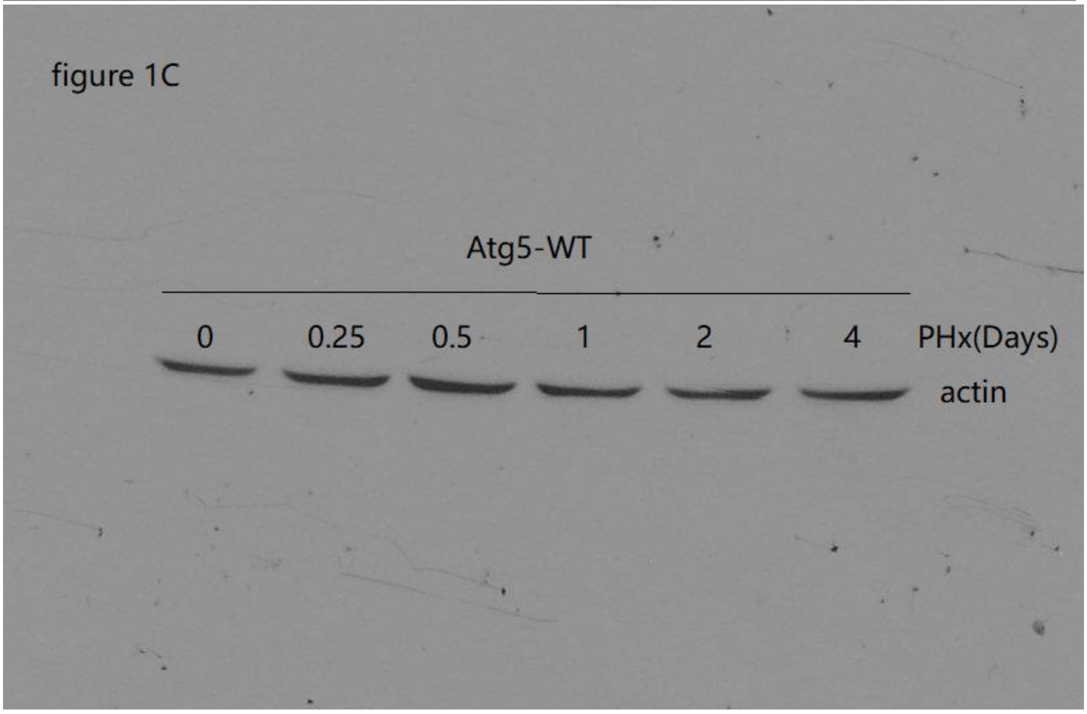
figure 1C
Atg5-WT
0
0.25
0.5
1
2
4
PHx(Days)
actin

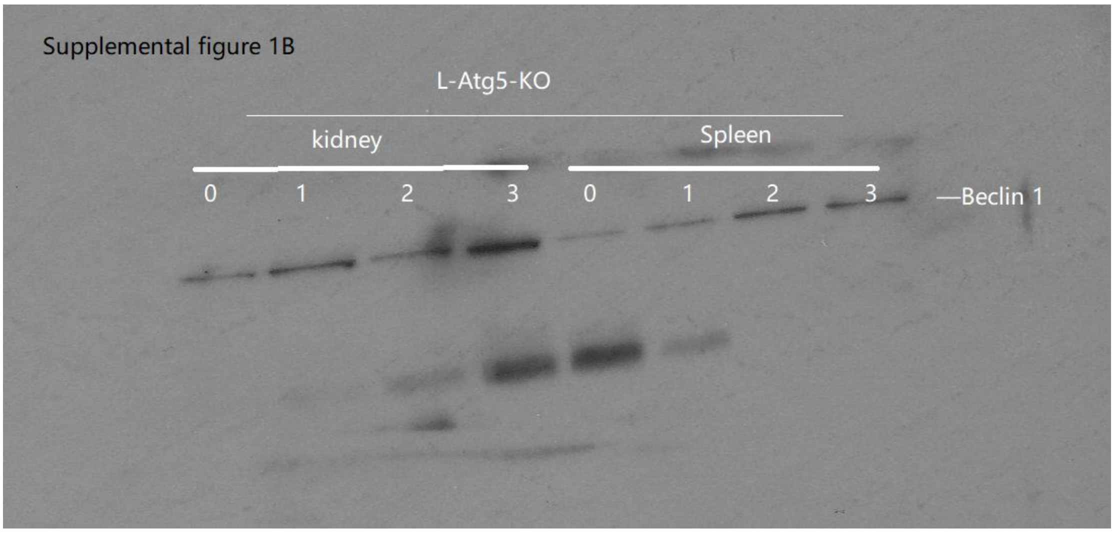
Supplemental figure 1B
L-Atg5-KO
kidney
Spleen
0
1
2
3
0
1
2
3
—Beclin 1

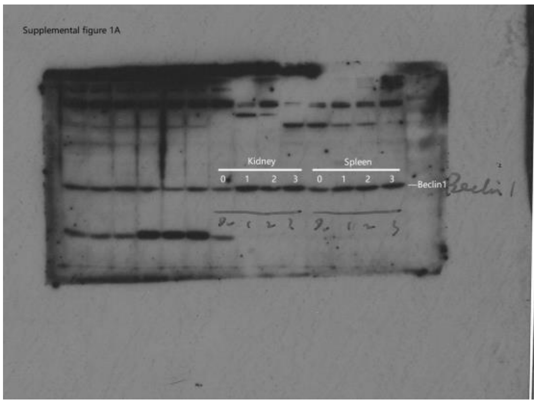
Supplemental figure 1A
Kidney
Spleen
0 1 2 3 0 1 2 3
—Beclin1
Beclin 1

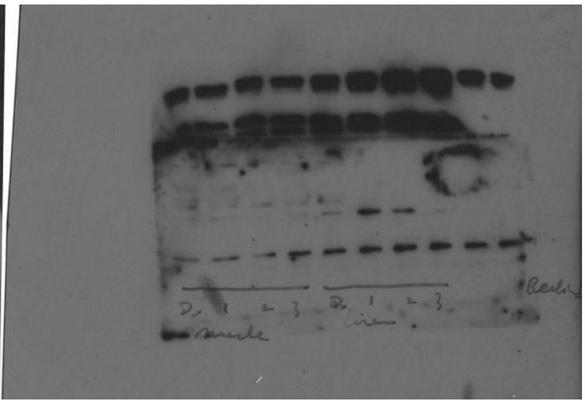

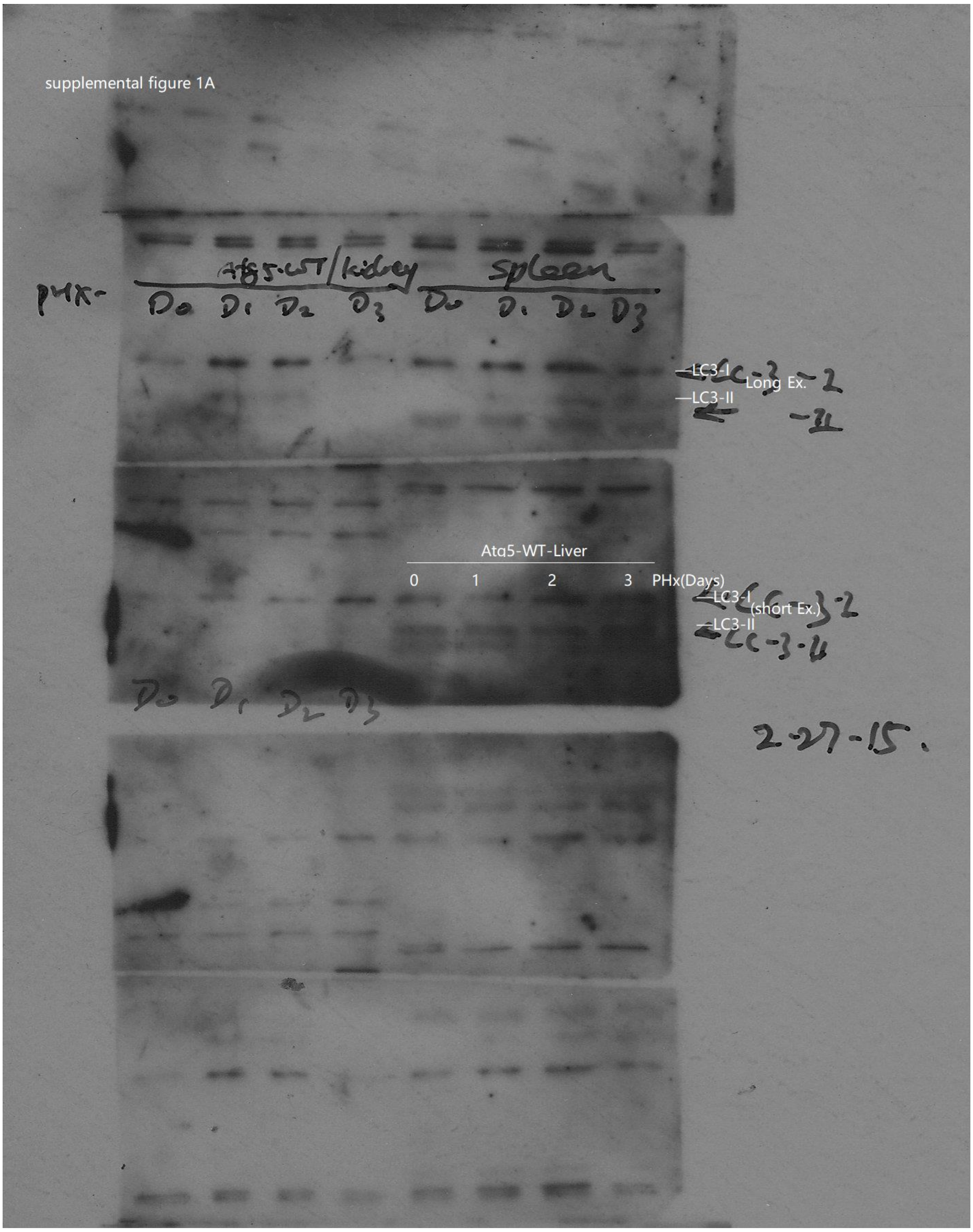
supplemental figure 1A
PHX-
spleen
D0 D1 D2 D3 D0 D1 D2 D3
—LC3-I
—LC3-II
Long Ex.
Atg5-WT-Liver
0 1 2 3 PHx(Days)
—LC3-I
(short Ex.)
—LC3-II
D0 D1 D2 D3
2-27-15.

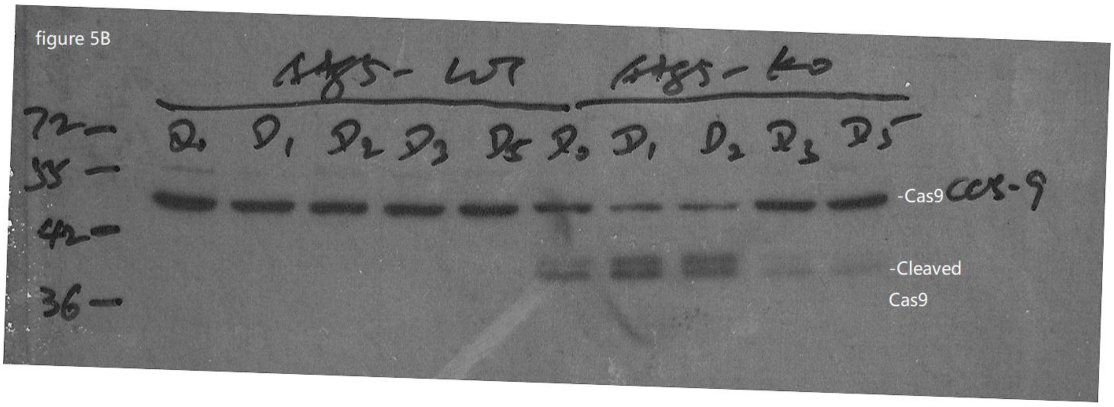
figure 5B
-Cas9
-Cleaved
Cas9

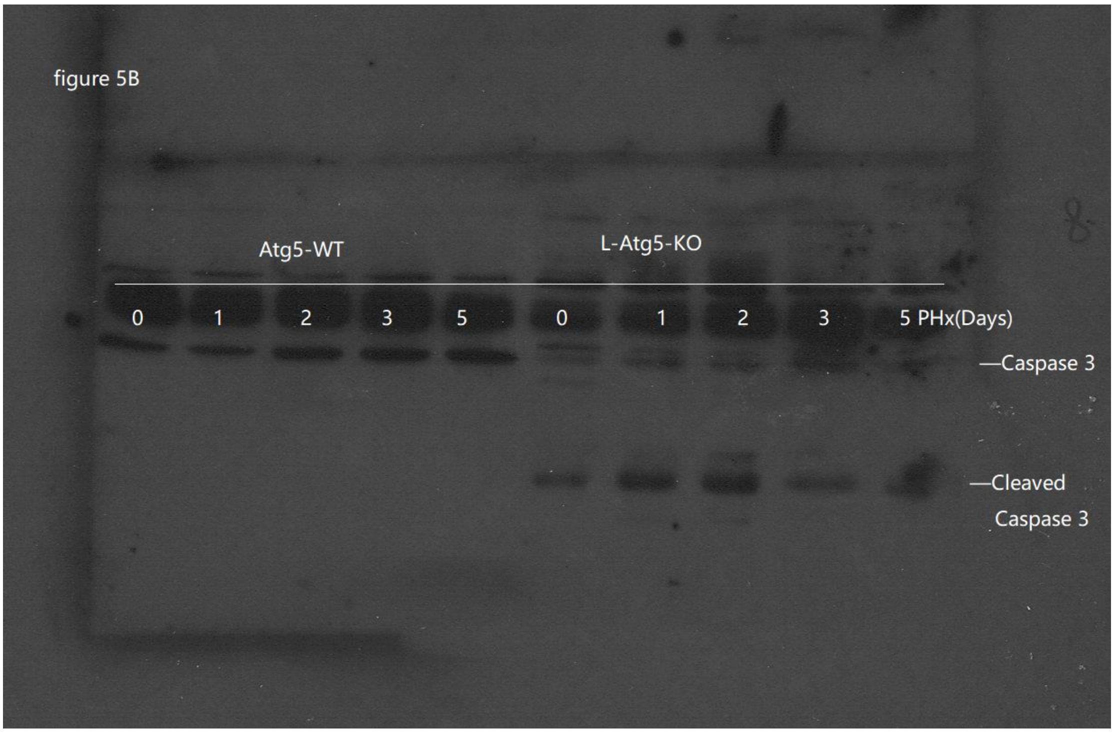
figure 5B
Atg5-WT
L-Atg5-KO
0 1 2 3 5 0 1 2 3 5 PHx(Days)
—Caspase 3
—Cleaved
Caspase 3

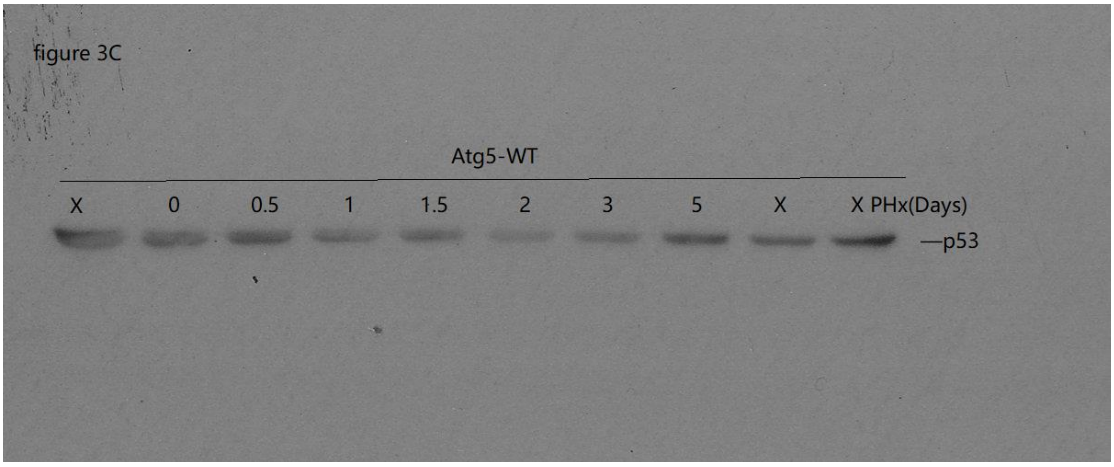
figure 3C
Atg5-WT
X 0 0.5 1 1.5 2 3 5 X X PHx(Days)
—p53

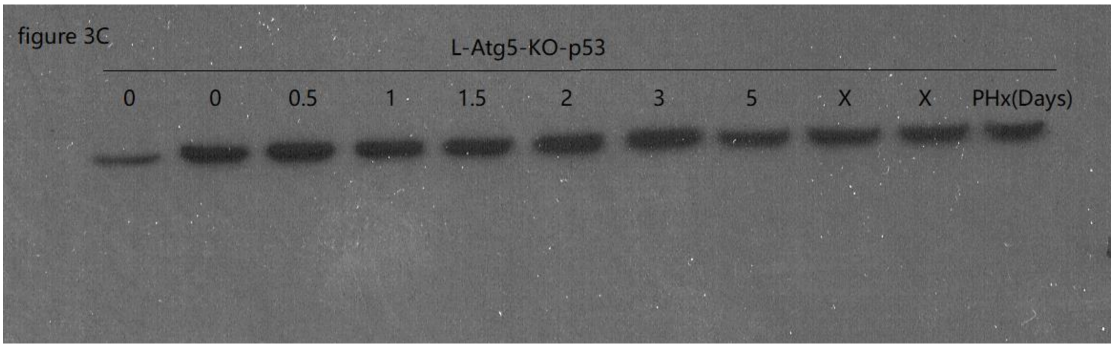
figure 3C
L-Atg5-KO-p53
0 0 0.5 1 1.5 2 3 5 X X PHx(Days)

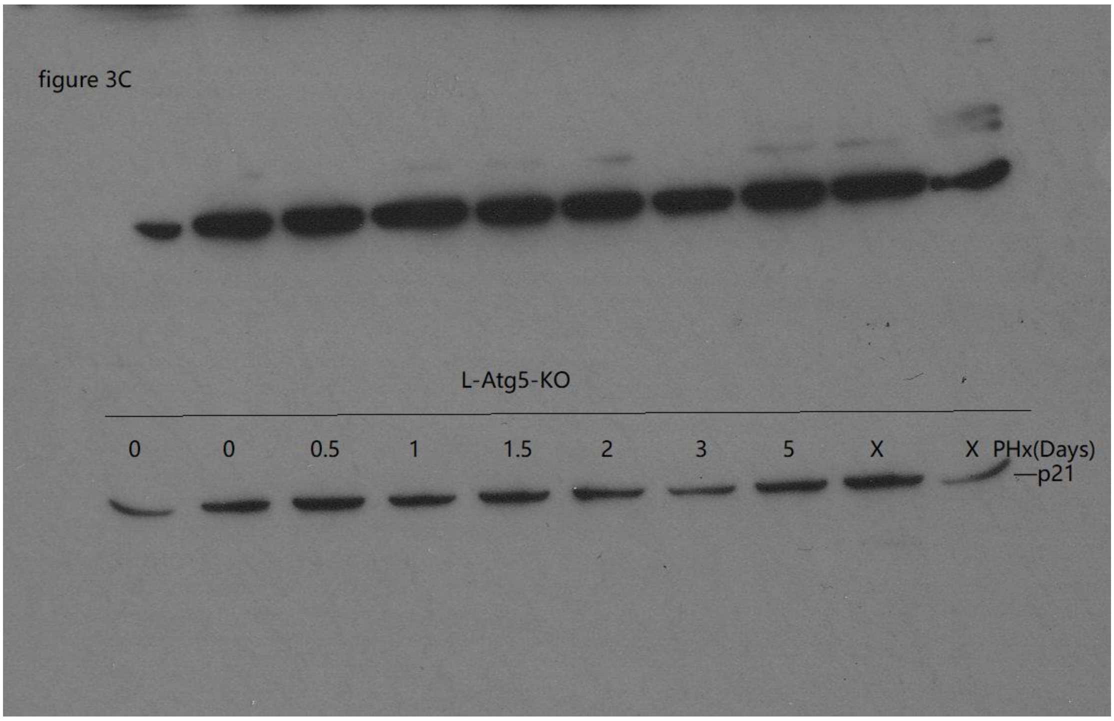
figure 3C
L-Atg5-KO
0 0 0.5 1 1.5 2 3 5 X X PHx(Days)
—p21

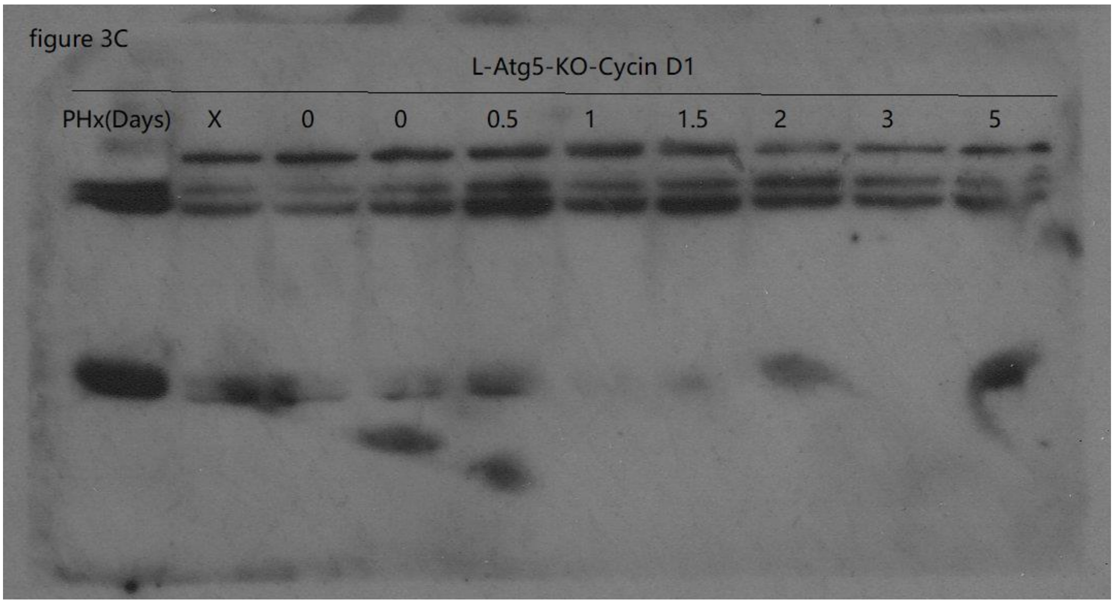
figure 3C
L-Atg5-KO-Cycin D1
PHx(Days) X 0 0 0.5 1 1.5 2 3 5

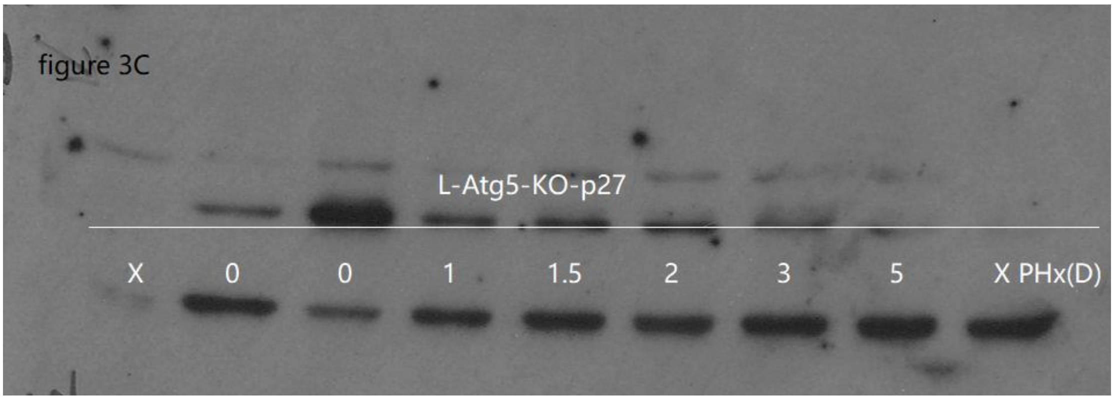
figure 3C
L-Atg5-KO-p27
X 0 0 1 1.5 2 3 5 X PHx(D)

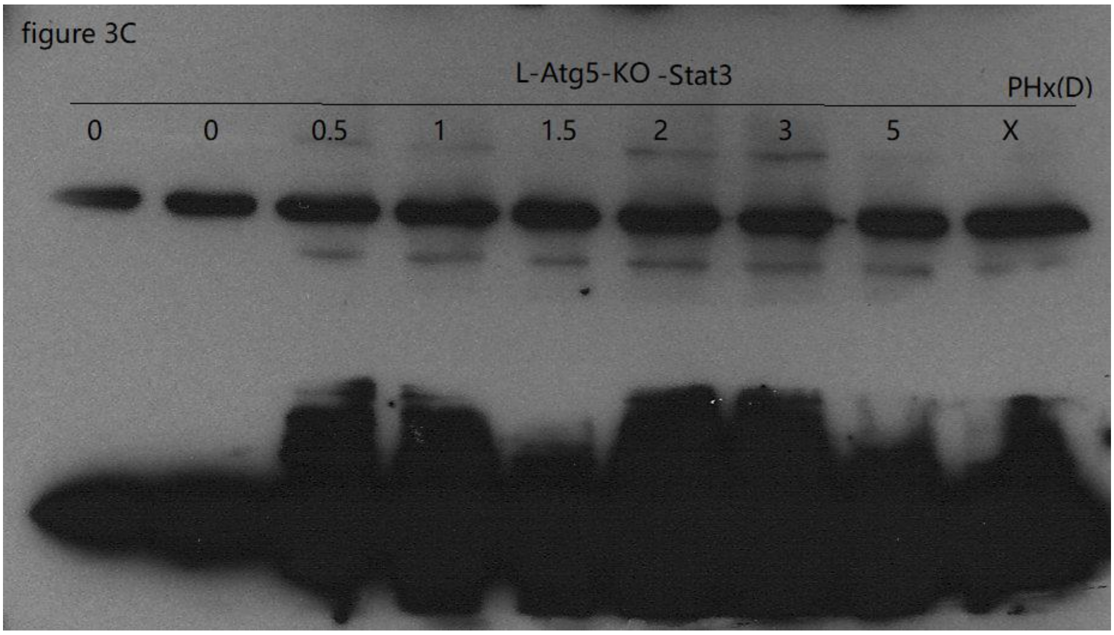
figure 3C
L-Atg5-KO -Stat3
PHx(D)
0 0 0.5 1 1.5 2 3 5 X

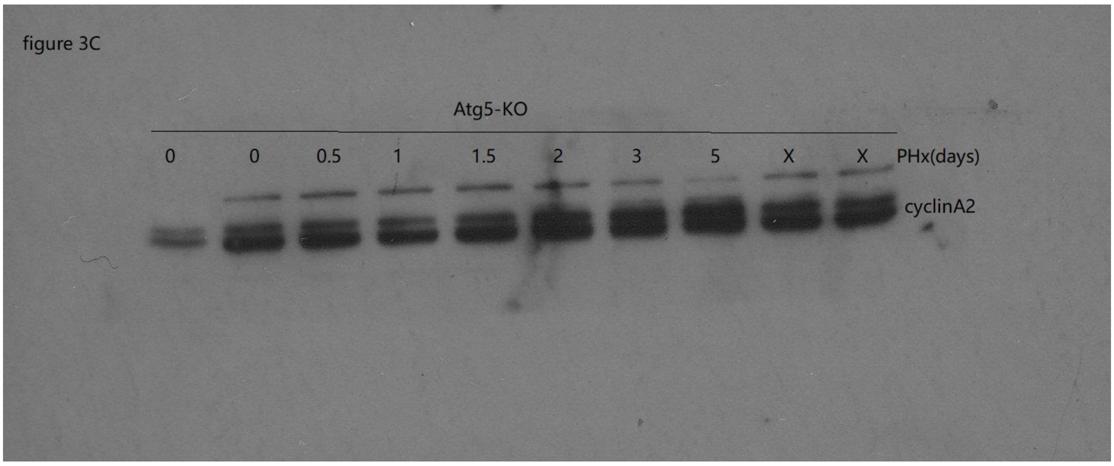
figure 3C
Atg5-KO
0 0 0.5 1 1.5 2 3 5 X X PHx(days)
cyclinA2

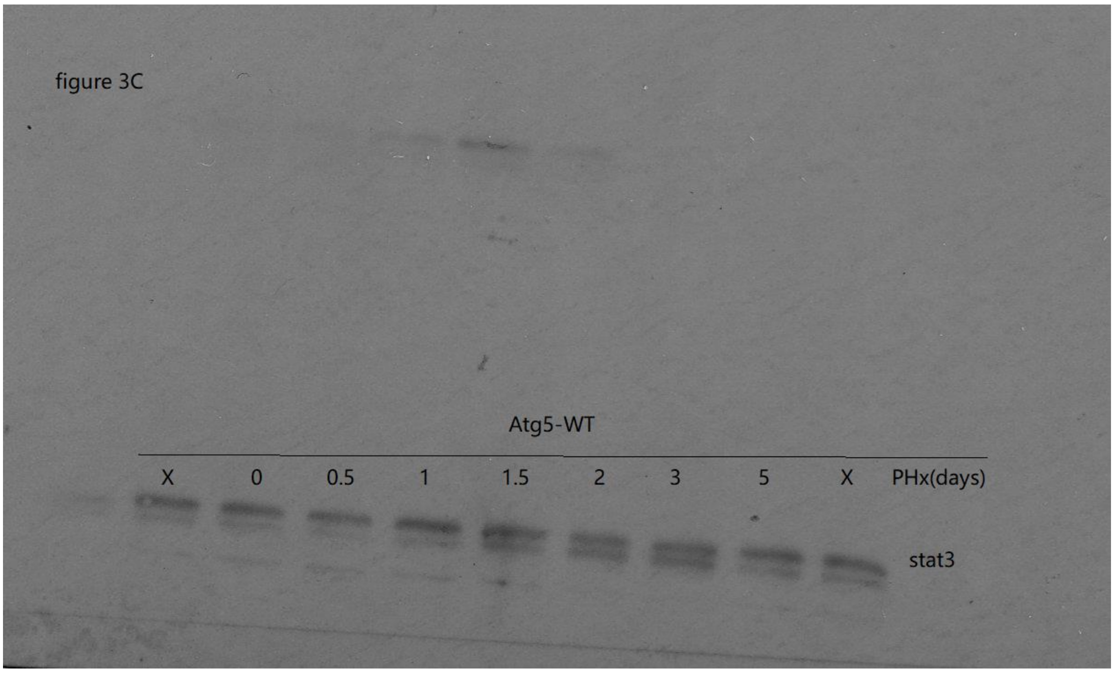
figure 3C
Atg5-WT
X 0 0.5 1 1.5 2 3 5 X PHx(days)
stat3

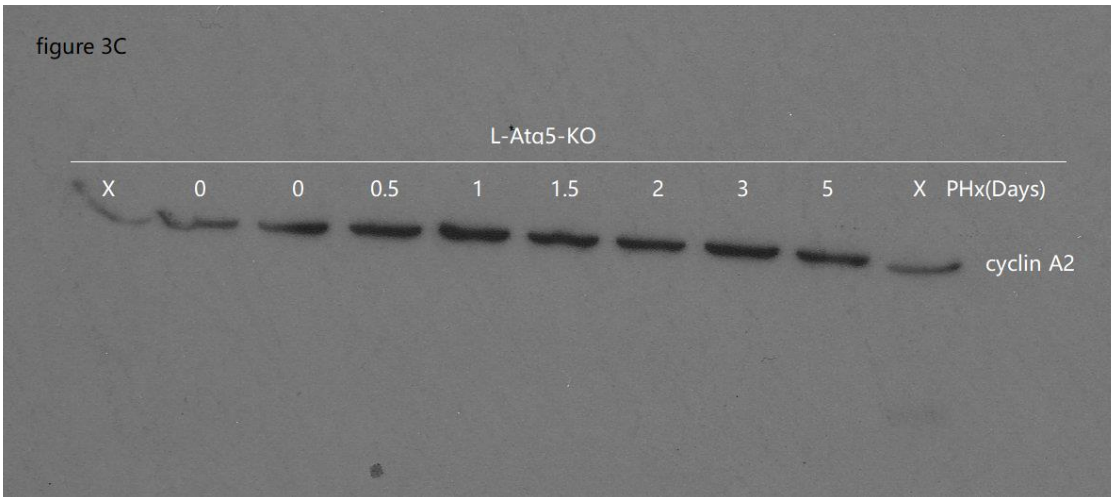
figure 3C
L-Atg5-KO
X 0 0 0.5 1 1.5 2 3 5 X PHx(Days)
cyclin A2

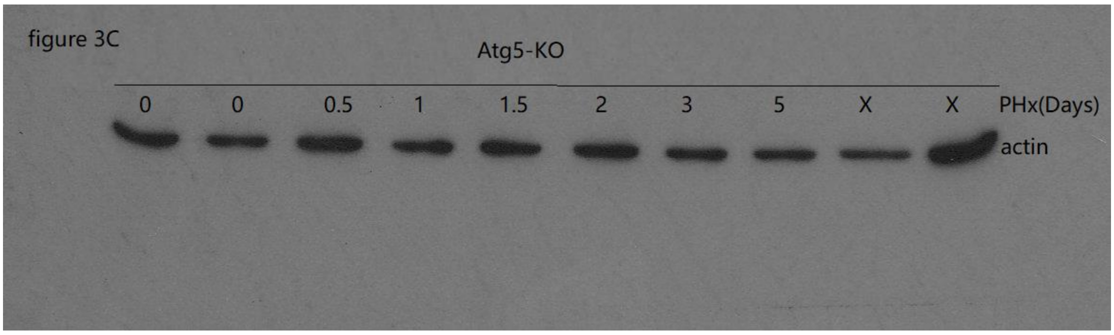
figure 3C
Atg5-KO
0 0 0.5 1 1.5 2 3 5 X X PHx(Days)
actin

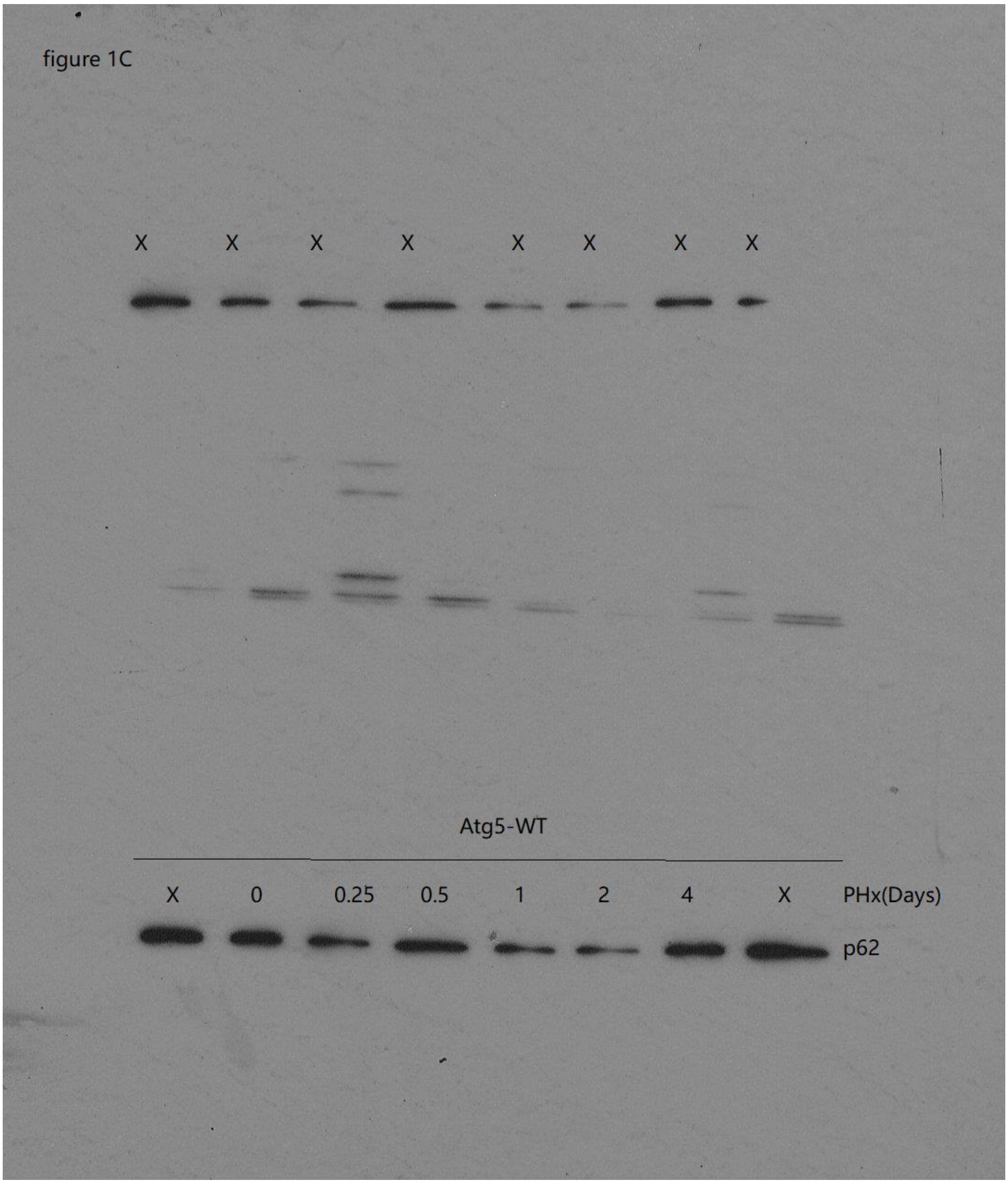
figure 1C
X X X X X X X X
Atg5-WT
X 0 0.25 0.5 1 2 4 X PHx(Days)
p62

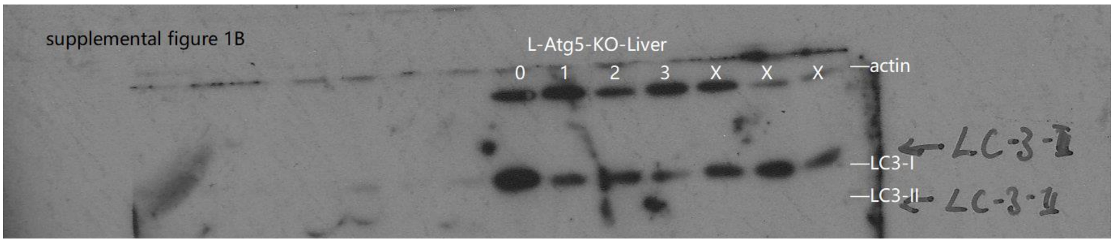
supplemental figure 1B
L-Atg5-KO-Liver
0 1 2 3 X X X
—actin
—LC3-I
—LC3-II
